# Utility of reticulocyte haemoglobin content and percentage hypochromic red cells as markers of iron deficiency anaemia among black CKD patients in South Africa

**DOI:** 10.1371/journal.pone.0204899

**Published:** 2018-10-03

**Authors:** Aishatu Muhammad Nalado, Johnny N. Mahlangu, Raquel Duarte, Graham Paget, Gbenga Olorunfemi, Barry F. Jacobson, Saraladevi Naicker

**Affiliations:** 1 Department of Internal Medicine, School of Clinical Medicine, Faculty of Health Science, University of the Witwatersrand, Johannesburg, South Africa; 2 Department of Internal Medicine, College of Health Sciences, Bayero University, Kano, Nigeria; 3 School of Pathology, Faculty of Health Sciences, University of the Witwatersrand, Johannesburg, South Africa; 4 Division of Epidemiology and Biostatistics, School of Public Health, University of the Witwatersrand, Johannesburg, South Africa; Universidade Estadual Paulista Julio de Mesquita Filho, BRAZIL

## Abstract

**Introduction:**

Iron deficiency anaemia (IDA) worsens the prognosis and outcomes of chronic kidney disease (CKD). However, while the haemoglobin level is unreliable for early detection of IDA, reticulocyte haemoglobin content (CHr) and hypochromic red cells (%HYPO) are early markers of IDA.

**Methods:**

This was a cross sectional study of black adult participants (n = 258) with CKD and apparently healthy members of staff and patients’ relatives (n = 141) at the Charlotte Maxeke Johannesburg Academic Hospital, South Africa, between 1 June 2016 and 31 December 2016. Serum iron, serum ferritin and transferrin were measured using standard laboratory methods, while the haematology analyser was employed to measure CHr and %HYPO. The validity of CHr and %HYPO as markers of IDA were evaluated. Multivariable binary logistic regression was conducted to determine predictors of the relationship between IDA, CHr and %HYPO. The area under the receiver operator characteristics (ROC) curve (AUC) of the final models were utilised to evaluate the discriminatory value of CHr and %HYPO respectively.

**Results:**

About one-quarter (26.1%) of the participants had IDA which was more than three times more frequent among CKD patients, compared to controls (35.3% vs 9.2%); 32.3% (95%CI: 27.90%– 37.10%) of the study population had iron deficiency without anaemia and the prevalence of iron deficiency without anaemia was lower in CKD patients compared to controls (29.5% vs 37.6%). The mean age of CKD patients was higher than in controls (52.7 ±14.3 vs 40.4 ±12.6 years, P-value<0.001). The sensitivity and specificity for diagnosing IDA among CKD participants was 62.6% and 80.2% respectively for CHr (at a cut-off value of <28pg) and 63.3% and 79.8% respectively for %HYPO. CKD participants with CHr levels >28pg were 82% less likely to be diagnosed as having IDA as compared to those with CHr levels ≤ 28pg) (adj odds ratio = 0.18, 95% CI: 0.09–0.37). The AUC of CHr (0.81, 95% CI: 0.76–0.87) was higher than the AUC of %HYPO (0.76, 95%CI: 0.70–0.82).

**Conclusion:**

The diagnostic usefulness of CHr and the screening performance of %HYPO in predicting IDA among CKD patients are high. Their lower cost compared to conventional markers of ID recommend their use in clinical practice. Further cost effectiveness studies of these parameters are warranted.

## Introduction

The prevalence of anaemia is high among chronic kidney disease (CKD) patients due to multiple factors [1, 2]. Anaemia also impacts on the morbidity and mortality of CKD patients by accelerating disease progression and decreasing survival [3]. The importance of anaemia prevention, monitoring, and management in CKD patients cannot be overemphasised, as an intricate balance must be maintained between stimulation of erythropoiesis and prevention of iron overload among CKD patients [4]. Moreover, treatment of iron deficiency anaemia (IDA) is an important component of care for CKD patients, with numerous benefits such as higher tolerance for physical activity, improved cognitive and cardiovascular function, better quality of life, and lower mortality [1,2].

Monitoring of haemoglobin (Hb) levels alone may not be adequate for evaluating IDA among CKD patients as the decline in haemoglobin levels occurs late in IDA [5]. Furthermore, changes in serum levels of traditional iron parameters such as iron, transferrin saturation, and ferritin may not always correlate with the functional iron deficiency (FID) status of the patient [5]. However, FID status is clinically relevant since it defines the instantaneous iron deficiency state of the patient and guides management [5]. Monitoring of FID status is also important among CKD patients who are on recombinant erythropoietin therapy [2].

Changes in the morphology and other indices of red blood cells and reticulocytes correlate with the dynamic state of iron deficiency (ID [1–6]. Hence, the evaluation of IDA among CKD patients (especially those on treatment for IDA) can be enhanced by monitoring their reticulocyte haemoglobin content (CHr) and percentage hypochromic red cell levels (%HYPO) [6–8]. CHr and %Hypo tests are 4 times cheaper than the conventional haematological tests and they can be used to detect ID before clinical manifestations of anaemia are observed [9]. CHr and %HYPO can also be reliable for detecting IDA among CKD patients because inflammatory conditions do not affect the levels of reticulocyte haemoglobin content.

Bone marrow iron stores are often regarded as the best indicator (gold standard) of iron status [10]. However, bone marrow biopsy is invasive and predisposes to the risk of infection, or bleeding at the puncture site [11]. Bone marrow biopsy procedure is also relatively costly [11]. Thus, a bone marrow biopsy is not generally advocated for routine monitoring of IDA in CKD patients. Determination of functional iron status involves measuring the proportion of %HYPO. %HYPO is an index that provides information about functional iron status several months before the manifestations of clinical anaemia are present and it is a late indicator of iron restricted erythropoiesis [12,8]. Likewise, CHr is an early marker of functional iron deficiency, as reticulocytes exist in the circulation for only 1–2 days. Thus, CHr can be useful in monitoring the early state of IDA and response to erythropoietin therapy [5]. Since classical laboratory biomarkers of ID exhibit wide biological variability in CKD patients [12], there is need to evaluate other novel and consistent markers. The validity of a screening or diagnostic tool is related to the disease prevalence and the attributes of the study population. Hence, it is not recommended to extrapolate results of the validity of a test of a study population to a different population. Furthermore, since anaemia severity also varies across the spectrum of CKD stages, the efficiency of markers of IDA may be affected by the CKD stage. Thus, we aimed to evaluate the usefulness of CHr and %HYPO in the diagnosis of IDA and their performance across the CKD stages, and compare them with other traditional markers of iron deficiency in pre-dialysis black CKD patients.

## Materials and methods

This cross-sectional analytical study was conducted at the Charlotte Maxeke Johannesburg Academic Hospital (CMJAH) from July to December 2016. The study was approved by the Human Research Ethics Committee of the University of the Witwatersrand. All the study participants signed informed consent prior to enrolment into the study.

Three hundred and ninety-nine adult participants were recruited for the study. The CKD cases (n = 258) were black non-dialysis requiring patients of the Renal Out-Patient Clinic of CMJAH while the comparator group were apparently healthy members of staff and patients’ relatives (n = 141). The inclusion criteria of the study were patients aged 18 years and above, with CKD as diagnosed based on estimated glomerular filtration rate (eGFR) <60ml/min. GFR was calculated using the CKD EPI formula [13]. Patients with active infection, malignancy, gastrointestinal bleeding, active inflammation, on immunosuppressant therapy, HIV infection, known haemoglobinopathies, and those who had received a blood transfusion in the three months preceding recruitment were excluded from the study.

Early morning venous blood samples were drawn from the patients. Biochemical iron status, serum iron, total iron binding capacity (TIBC), and serum ferritin were measured. Serum iron was determined by ferrozine calometric method, TIBC by colorimetric chromazurol dye binding method using ADVIA 1800 (Siemen Medical Solutions Diagnostic, USA), and serum ferritin was determined by using two-site chemiluminescent immunometric assay by (Siemens Medical Solutions Diagnostics, USA). Transferrin saturation was calculated by the formula: serum iron/ TIBC. Complete blood counts, CHr and %HYPO were obtained after processing the blood samples using the Siemens ADVIA 2120, Technion H3 RTX and RTC system analyzer (Siemens Medical Solutions Diagnostics, Tarrytown, NY).

Haematological deficiencies were defined based on the internationally accepted iron parameters for CKD patients [2]. Thus, based on the Kidney Disease Outcomes and Quality Initiative target-to-treat (KDOQI) cut-offs and National Institute for Health and Care Excellence (NICE) guidelines, absolute iron deficiency was defined as serum ferritin <100μg/L and transferrin saturation (TSAT) <20% [14, 15]. Functional iron deficiency was defined as serum ferritin level of >100ug/L and TSAT <20%. Iron deficiency anaemia was defined as TSAT <20%, ferritin <100μg/L, and Hb <12g/dl in women, and <13g/dl in men [2, 5, 15]. Although the Kidney Disease Improving Global Outcomes (KDIGO) defined iron deficiency as TSAT<30% and ferritin <500ug/L[16, 17], this definition was not utilized in this study. Prevalence of iron deficiency without anaemia (with Hb >12g/dl in women, and >13g/dl in men) was also ascertained in our study population.

### Statistical analysis

Continuous variables with normal distribution were reported as means ± standard deviations and non-normally distributed continuous variables were reported as medians and interquartile ranges. Categorical variables were described as frequencies and percentages. The correlation of each parameter with other haematological indices was performed using Spearman correlation coefficients. Among the CKD participants, the specificity, sensitivity, negative predictive value and positive predictive values of CHr (at cut-off <28pg) and %HYPO (at cut-off >5%), as markers of IDA were evaluated using TSAT and ferritin as the reference. Multiple logistic regression analysis was used to determine the association between CHr and IDA; %HYPO and IDA; and combined CHr/%HYPO and IDA among the CKD participants. Variables with P-values <0.2 on univariable analysis were eligible for inclusion into the multivariable models. Thus, three models were built (one each for: CHr and IDA; %HYPO and IDA; and combined CHr/%HYPO and IDA). Receiver operator characteristics (ROC) curves as well as the area under the curve (AUC) of each of the final models were utilised to further evaluate the discriminatory value of CHr; %HYPO and a combination of CHr and %HYPO in diagnosing IDA. Comparison of the performance of CHr, %HYPO and their combination in predicting anaemia was conducted by stage of CKD using the AUC values. Association between gender and CKD stages was assessed with Pearson’s Chi-square test. P-value < 0.05 was taken as the level of statistical significance and we assumed a two-tailed test of hypothesis. All statistical analysis was performed using STATA 14.0 software (Stata Corp, USA)

## Results

### Study participants

**Fig 1:**
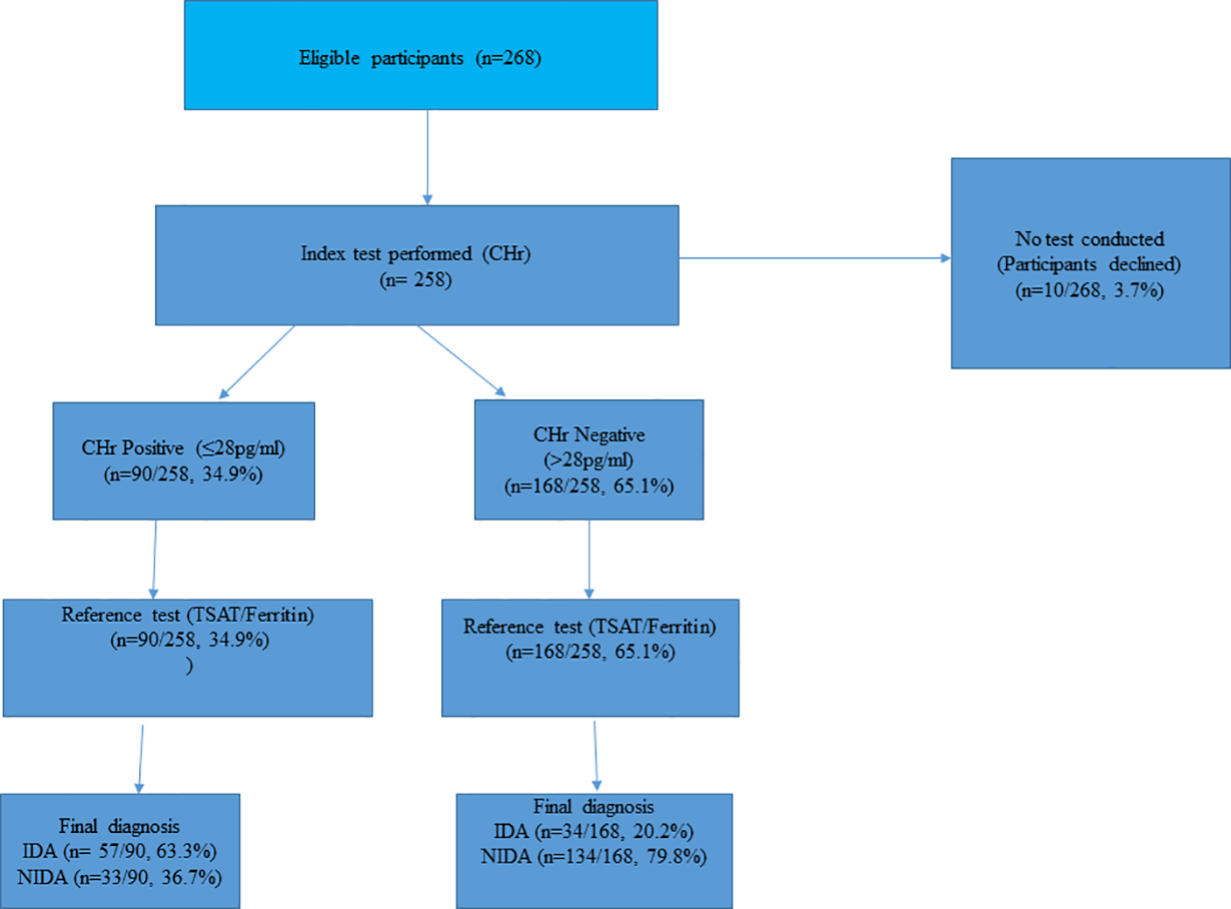
Flow chart of study participants.

Of the 258 CKD participants,34.9% (n = 90/258) had CHr levels ≤28pg/ml. Fig 1 below showed the flow chart of the study participants.

Compared to the controls, the CKD patients were older, had higher mean systolic blood pressure, significantly lower Hb, and higher serum ferritin levels. Similar proportions of CKD patients were found in stages 3b, 4 and 5; about one-fifth of the CKD patients were in stages 3b, 4 and 5 each respectively (Table 1).

**Table 1:** Comparison of the socio-demographic, haematological and biochemical characteristics of CKD patients and controls.

Characteristics	CKD patients	Controls	Total	P-value
**Age (years)**				
Mean (±SD)	52.7 (±14.3)	40.4 (±12.6)	48.3 (±14.9)	<0.0001
<25	6 (2.3)	18 (12.8)	24 (6.0)	<0.0001
25–34	22 (8.5)	34 (24.1)	56 (10.0)	
35–44	43 (16.7)	43 (30.5)	86 (21.6)	
45–54	65 (25.2)	23(16.3)	88 (22.1)	
55–64	69 (26.7)	16 (11.4)	85 (21.3)	
≥ 65	53 (20.5)	7 (5.0)	60 (15.0)	
**Gender, n (%)**				
Male	134 (51.9)	59 (41.8)	193 (48.4)	0.054
Female	124 (48.1)	82 (58.2)	206 (51.6)	
**Stage of CKD**				
stage1/normal	23 (8.9)	97 (68.8)	120 (30.1)	<0.001
stage 2	34 (13.2)	43 (30.5)	77 (19.3)	
stage 3a	32 (12.4)	1 (0.71)	33 (8.3)	
stage 3b	52 (20.2)	0 (0.0)	52 (13.0)	
stage 4	59 (22.9)	0 (0.0)	59 (14.8)	
stage 5	58 (22.5)	0 (0.0)	58 (14.5)	
**Systolic Blood pressure (mmHg)**				
Mean (SD)	143.6 (± 22.8)	135.2 (± 11.3)	140.6 (± 19.9)	<0.0001
**Diastolic Blood pressure (mmHg)**				
Mean (SD)	81.8 (± 15.55)	82.2 (± 10.4)	82.0 (± 13.9)	0.78
**Haemoglobin, Hb (g/dl)**				
Mean (SD)	12.2 (±2.7)	14.1 (±1.8)	12.9 (±2.6)	<0.0001
Reticulocyte Hb				
Median (IQR)	29.33(27.66–31.22)	30.2(28.6–31.2)	29.88(27.88–31.22)	0. 0.0256
**Hypochromic red cells**				
Median (IQR)	7.7(3.1–14.2)	3.8 (1.9–5.9)	5.3 (2.3–12.3)	<0.0001
^**#**^**MCHC**				
Mean (SD)	32.4 (± 1.9)	32.6 (± 1.3)	32.4 (± 1.9)	0.307
**MCV**				
Mean (SD)	87.8 (± 6.3)	88.2 (± 5.2)	87.9 (± 5.9)	0.4788
**Serum Ferritin**				
Median (IQR)	103 (58–197)	76.0 (56–144)	92 (56–177)	0.0015
^**TSAT**				
Median (IQR)	18 (13–22)	20 (16–24)	18 (14–23)	0.0021
**eGFR(ml/min)**				
Mean (SD)	41.2 (±35.0)	99.6 (±18.7)	61.8(±41.2)	<0.0001

^TSAT: Transferrin saturation; eGFR: estimated Glomerular filtration rate;

^#^MCHC: Mean corpuscular haemoglobin concentration;

MCV: Mean corpuscular volume; IQR: Inter-quartile range

The overall prevalence of IDA was 26.1% (95%CI: 21.98% - 30.62%) and was more than three times higher among CKD participants as compared to the control group (35.3% vs 9.2%), as shown in Table 2. The proportion of functional iron deficiency anaemia (18.6% vs 1.4%) and absolute iron deficiency anaemia (16.7% vs 7.8%) was higher among CKD patients, compared to controls However, 32.3% (95%CI: 27.90%– 37.10%) of the study population had iron deficiency without anaemia and the prevalence of iron deficiency without anaemia was lower in CKD patients compared to controls (29.5% vs 37.6%). (data not shown)

**Table 2:** Iron status in the study participants.

	CKD n (%)	Controls n (%)	Total	P-value
Iron deficiency anaemia	91 (35.3)	13 (9.2)	104(26.1)	<0.001^#^
Functional iron deficiency anaemia	48 (18.6)	2 (1.4)	50 (12.5)	<0.001^
Absolute iron deficiency anaemia	43 (16.7)	11 (7.8)	54 (13.5)	0.013^#^

^Fischer’s exact test

^#^ Chi-square

There was a moderate positive correlation between haemoglobin levels and CHr levels among CKD patients (α = 0.56, P<0.0001). While MCV and MCHC levels showed a weak positive correlation with CHr among the CKD patients, %HYPO demonstrated a moderate (α = -0.42, P<0.0001) and weak negative correlation with MCHC and Hb respectively, among CKD patients (Table 3).

**Table 3:** Correlation of reticulocyte haemoglobin content and percentage hypochromic red cells with other haematological parameters among CKD participants and healthy controls.

Haematological parameters	Chronic Kidney Disease patients				Healthy Controls			
	Hypochromic level		CHr		Hypochromic level		CHr	
	α	P-value	α	P-value	α	P-value	α	P-value
HB	-0.27	<0.0001	0.56	<0.0001	-0.30	0.0004	0.18	0.0372
MCV	0.009	0.8999	0.33	<0.0001	-0.42	<0.0001	0.59	<0.0001
MCHC	-0.42	<0.0001	0.38	<0.0001	-0.54	<0.0001	0.37	<0.0001

α: Spearman’s rank correlation

Table 4 shows the sensitivity and specificity of diagnosing IDA among CKD participants. The sensitivity and specificity of CHr levels at a cut-off <28pg were 62.6% and 80.2% respectively. The positive predictive and the negative predictive values of CHr levels among CKD patients were 63.3% and 79.8%, respectively. However, the sensitivity and specificity of diagnosing IDA among CKD patients using %HYPO (>5%) was 73.6% and 44.3% respectively. The positive predictive and the negative predictive values of %HYPO (>5%) among CKD patients were 41.9% and 75.5%, respectively.

**Table 4:** Predictive ability of reticulocyte haemoglobin content and percentage hypochromic red cells using TSAT and ferritin as reference parameters among chronic kidney disease participants and healthy controls.

		CKD patients		Total	Healthy Controls		Total
		NIDA	IDA		NIDA	IDA	
CHr n (%)	≤28pg	33(19.76)	57(62.64)	90 (34.88)	22(17.19)	6 (46.15)	28 (19.86)
	>28pg	134(80.24)	34(37.36)	168(65.12)	106(82.81)	7(53.85)	113(80.14)
%HYPO	>5%	93 (55.69)	67(73.63)	160(62.02)	34 (26.56)	9 69.23)	43 (30.50)
	≤5%	74 (44.31)	24(26.37)	98(37.98)	94 (73.44)	4 (30.77)	98 (69.50)
	Total	167	91	258	128	13	141

IDA: Iron deficiency anaemia; NIDA: Non-iron deficiency anaemia

#### Univariable and multivariable model and receiver operator characteristics curves of CHr and %HYPOs in determining iron deficiency anaemia

On univariable analysis the CHr, %HYPO, gender, age and stage of CKD were significantly associated with iron deficiency anaemia (P-value <0.05).

After adjusting for potential confounders among the CKD patients (viz. gender, age, stage of kidney disease), participants with CHr levels >28pg were 82% less likely to be diagnosed as having IDA compared to those with CHr levels ≤ 28pg/ml) (adjusted odds ratio = 0.18, 95% CI: 0.09–0.37, P<0.001). Similarly, with the use of the %HYPO criteria cut off value of ≤5%, CKD patients with %HYPO criteria ≤5% were 48% less likely to be diagnosed as having IDA as compared to patients with %HYPO >5% (adjusted odds ratio = 0.52, 95% CI: 0.28–0.96, P<0.037). Male gender and participants with advanced CKD (stages IV and V) had 2-fold and 2.6 to 4.4-fold increased odds of developing IDA as compared to female gender and patients in early stages of CKD respectively (Table 5).

**Table 5:** Relationship between reticulocyte haemoglobin content, percentage hypochromic red cells and iron deficiency anaemia among chronic kidney disease participants.

	^1^Multivariable logistic regression analysis for CHr			^2^Multivariable regression analysis for %HYPO		
Factors	OR	95% CI	P-value	OR	95%CI	P-value
**CHr**						
≤ 28 (ID)	1.00	Ref	Ref	-	-	-
>28 (NID)	0.18	0.09–0.37	<0.001	-	-	—
**Percentage hypochromic red cells**						
>5% (ID)				1.00	Ref	Ref
≤5% (NID)				0.52	0.28–0.96	0.037
**Gender**						
Male	1.00	Ref	Ref	1.00	Ref	Ref
Female	2.12	1.16–3.89	0.015	2.25	1.27–4.01	0.006
**Age group (years)**						
<25	1.00	Ref	Ref	1.00	Ref	Ref
25–34	0.25	0.04–1.63	0.147	0.29	0.05–2.11	0.192
35–44	0.15	0.03–0.88	0.036	0.21	0.04–1.35	0.069
45–54	0.20	0.04–1.06	0.058	0.26	0.06–1.48	0.101
55–64	0.86	0.18–4.22	0.855	1.06	0.25–6.04	0.941
65 and above	0.41	0.08–2.03	0.272	0.37	0.08–2.03	0.230
**Stage of kidney disease**						
Early stage	1.00	Ref	Ref	1.00	Ref	Ref
Late stage	2.64	1.34–5.21	0.005	4.35	2.36–8.03	<0.001

^1^Multivariable logistic regression of the model of the relationship between CHr and iron deficiency anaemia (CHr only).

^2^Multivariable logistic regression of model of the relationship between %HYPO and iron deficiency anaemia (%HYPO only).

The 2 models corrected for gender, age, stage of disease. OR: Adjusted odds ratio. Early stage: Stages 1–3; Late stage: Stages 4 and 5.ID: Iron deficiency NID: Non-iron deficiency.

The combined model of CHr and %HYPO (“S1 Table”) showed that while the odds ratio of CHr did not change significantly (adjusted odds ratio changed from 0.18 in Table 5 to 0.2 in S1 Table), the odds ratio for %HYPO was not significant.

There was no statistically significant relationship between gender and CKD stages (P-value = 0.101) (S2 Table)

#### Comparison of the performance of CHr and %hypochromic red cells in diagnosing iron deficiency anaemia among Chronic Kidney disease participants

**Fig 2:**
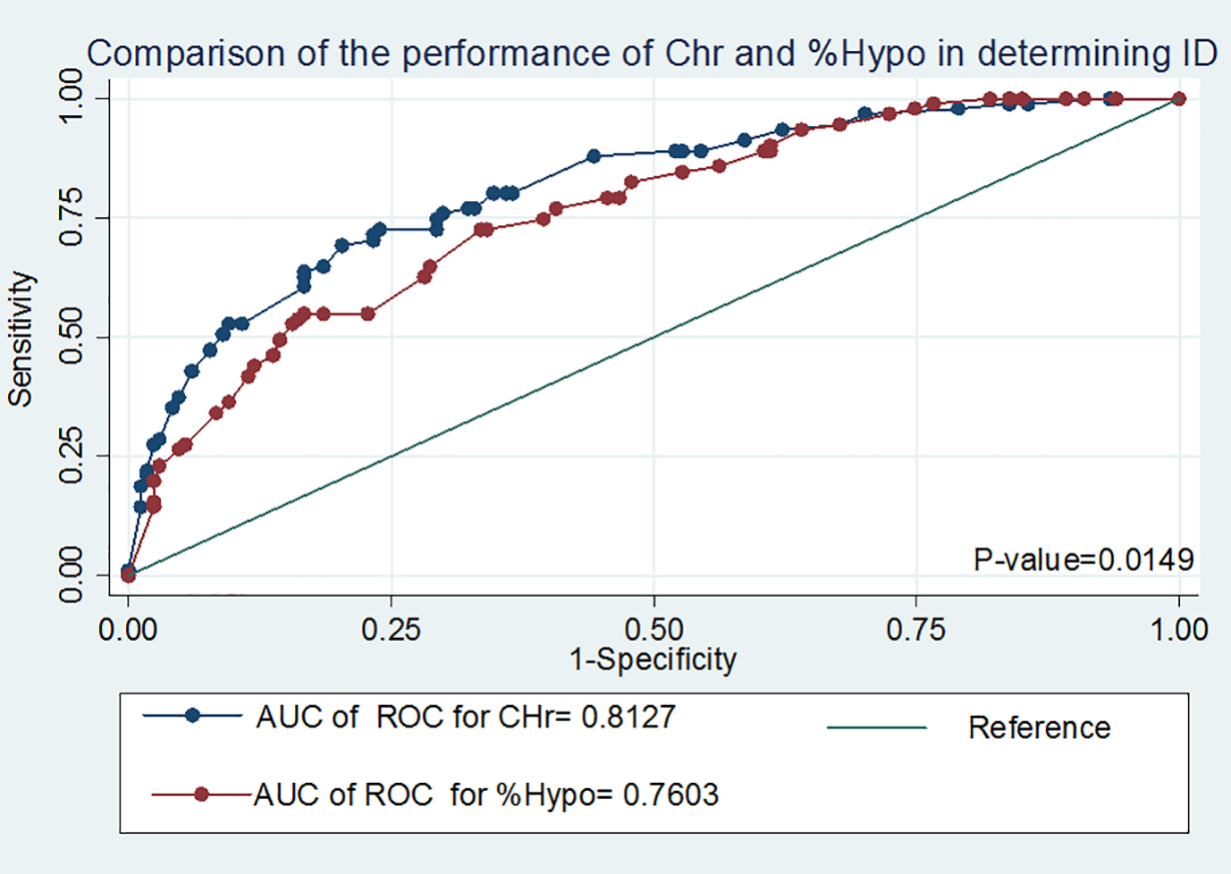
Comparison of the receiver operator characteristics (ROC) of reticulocyte haemoglobin content (CHr) and percentage hypochromic red cells.

The AUC of the ROC curve was higher for CHr as compared to %HYPO (81.3%% vs 76.0%, P = 0.0149) in diagnosing IDA (Table 6, Fig 2) below. Thus, CHr has higher discriminatory value for diagnosing IDA as compared to %HYPO. There was no additional value in combining CHr and %HYPO in diagnosing IDA as compared to utilising only CHr (P-value = 0.94) in diagnosing IDA among our cohort. In addition, the CHr performed better in the early stages of CKD (stages I and II) as compared to the later stages (IV and V) (82% vs 76%) of CKD.

**Table 6:** Performance of reticulocyte haemoglobin content and percentage hypochromic red cells in the diagnosis of iron deficiency anaemia in chronic kidney disease participants.

Parameter	AUC of ROC	(95%CI)
**CHr**	0.81	0.76–0.87
**Hypochromic red cells**	0.76	0.70–0.82
**CHr + Hypochromic red cells**	0.81	0.76–0.87
**CHr by stage**		
Early	0.82	0.72–0.84
Late	0.76	0.70–0.82
**Hypochromic Red cells by stage**		
Early	0.77	0.70–0.82
Late	0.72	0.58–0.72
**CHr+Hypochromic Red cells by stage**		
Early	0.85	0.71–0.84
Late	0.76	0.68–0.81

AUC: Area under curve. ROC: Receiver operator characteristics curve 95%CI: 95%: Confidence interval. Comparison of performance: CHr Vs % HYPO, P-value = 0.0149, CHr Vs (CHr+%HYPO), P-value = 0.94; %Hypo Vs (CHr+%HYPO), P-value = 0.0162

## Discussion

Over the past few decades, studies from developed countries have assessed the diagnostic utility of non-conventional iron markers such as CHr and %HYPO in predialysis and dialysis patients. However, little is known about the performance of these tests in African CKD populations. Hence, we evaluated the validity and performance of CHr and %HYPO in diagnosing IDA among black CKD patients to provide evidence for their utility in a low resource clinical setting like ours.

We found that the sensitivity and specificity of CHr, at a cut-off of 28pg, was 62.6% and 80.2% respectively [10]. This finding was higher than that of Fishbane et al. [10, 11], who reported a specificity of 75.5% and a relatively lower sensitivity of 41.9% with the use of CHr in identifying IDA among patients on maintenance haemodialysis. The possible explanation for this finding could be attributed to the relatively long lifespan of erythrocytes; since information about the status of iron stores in the body over a long period of time can be obtained from changes in erythrocytes levels, CHr can potentially be useful in early detection of chronic IDA [11]. Diagnostic tools should normally have high specificity so that positive cases can be determined with high certainty. Our study revealed that the specificity of CHr was high (80.2%) and participants with CHr levels >28pg were 82% less likely to be diagnosed with IDA as compared to those with CHr levels ≤ 28pg; in other words, those diagnosed with IDA at a cut-off of 28pg had about 5.6-fold odds of being diagnosed with IDA as compared to those with a higher CHr level. Therefore, CHr can be safely considered for use as a diagnostic tool for IDA in our study population. In addition, we found that the performance of a combination of %HYPO and CHr in detecting IDA was not superior to CHr alone. Thus, the single less costly test of CHr can be safely utilised for diagnosis and monitoring of IDA in our environment.

We further assessed and compared the predictive ability of CHr in detecting IDA across the stages of CKD and found a better performance of CHr in early stages of CKD than in later stages of CKD (82%vs 76%), and this difference was statistically significant (p = 0.0162). To our knowledge, this is the first time a relationship between CHr and its performance in diagnosing IDA, according to stages of CKD is being evaluated. Our result may partly be explained by the fact that IDA is more frequent in early stages of CKD than in the later stages. Our findings was at variance with those of Aoun et al[17], who reported significant difference of iron deficiency by gender across different stages of CKD. Differences in our findings could be explained by differences in the studied populations. However, further studies are required to further explore this finding. Our study highlights the fact that there may be a need to utilise different cut-off values of CHr levels at different stages of CKD.

Some of the advantages of the CHr include its ability to provide a snap shot of the iron available for erythropoiesis, which can be used for early detection of IDA [11]. It is also cheaper than measurement of conventional iron markers. From our experience, a unit cost of running CHr is approximately $5- $7 US dollars, as compared to $25- $30 dollars for the cost of TSAT and ferritin. Therefore, CHr can serve as an alternative to conventional markers (TSAT and ferritin) of iron status in evaluating iron status in resource poor countries.

Additionally, CHr is free from the biological variability that affects serum iron, ferritin, and other biochemical parameters. CHr also reflects the iron that is made available at the time the reticulocyte was produced in the bone marrow. CHr levels also highlight abnormalities that are related to the reticulocyte stage of erythropoiesis which may be missed because of the short or transient nature of the reticulocyte stage [12,13].

Consistent with other studies, the prevalence of IDA in our CKD population was more than three-fold higher than the prevalence in the control group (35% versus 9%) [10]. Thus, anaemia remains a major source of morbidity in our cohort. Previous studies such as The National Health and Nutritional Examination Survey (2004) also reported a high prevalence of iron deficiency, occurring in 58% of men and 72% of women with CKD [18]. The Dialysis Outcomes and Practice Pattern Study (DOPPS) 2003 reported that iron deficiency was present in 31–38% of CKD patients on haemodialysis [19]. Another study reported the prevalence of IDA in pre-dialysis CKD to be 29% [20].

The use of different CHr cut–off values in different studies for discriminating iron deficiency anaemia in both non-dialysis and dialysis populations has led to variations in the specificities and sensitivities of this marker of ID. For example, both Thomas et al. and Fishbane et al. defined functional iron deficiency as CHr <28pg [12, 21],and reported that a CHr cut off level of <28pg predicted IDA better than serum ferritin and transferrin saturation among a cohort of dialysis patients on erythropoietin therapy[12, 21]. However, Kim et al. reported a cut off value of 32pg for prediction of IDA among haemodialysis patients [22]. Therefore, interpretation and comparison of the predictive ability of CHr should be done in the context of ethnic variations, study populations and whether participants were on erythropoietin therapy.

A study of non-CKD hospitalised patients in South Africa also used CHr to predict iron deficiency as opposed to other parameters, such as bone marrow aspirates. They established that a CHr of <28pg can predict IDA with a sensitivity of 75.8% and specificity of 84.1%, which was similar to our findings [23]. Another study at Pelonomi Hospital, South Africa, among infants and children showed the optimal CHr cut-off for the diagnosis of IDA to be 29pg, corresponding to a sensitivity of 86% and a specificity of 50%. This cut-off value was slightly higher than our cut-off value of 28pg, and this variation could be due to differences in age among the study population [24].

Consistent with some previous studies, we found that the sensitivity of using CHr to predict iron deficiency was equivalent to the conventional parameters (TSAT, ferritin) [14,15,16]. The further advantage of CHr, which includes its lower and affordable cost, makes it more attractive in low resource settings. Additionally, CHr is not influenced by inflammation and infection.

### Study limitations

Our results should be interpreted in the context of the limitations of the study. Our reference was serum ferritin/TSAT instead of bone marrow aspirate. However, we believe that our results are valid and would contribute to the growing literature on the subject. Bone marrow aspiration was not feasible, due to its cost and invasive nature.

Some diagnostic limitations may exist with CHr, as not all haematology analysers are capable of measuring CHr and % hypochromic red cells. Furthermore, CHr can be falsely low in cases of haemoglobinopathies causing microcytic anaemias, or falsely high in megaloblastic anaemias, though this group of patients was excluded from our current study population.

## Conclusion

CHr appears to be a useful tool to predict IDA among black non-dialysis CKD patients. There was no added advantage of combining the test with %HYPO in the diagnosis of IDA. Further studies are required to confirm the usefulness of CHr in this group of patients to improve practice. Furthermore, CHr could be a potential parameter to monitor early response to intravenous iron supplementation in pre-dialysis patients.

## Supporting information

S1 Checklist STARD checklist for the reporting of studies of diagnostic accuracy. (DOC)

S1 Table Combined model of performance of CHr and %HYPO. (DOC)

S2 Table Association between gender and stage of kidney disease among non-dialysis CKD patients. (DOC)

S1 Data (DTA)
