# Supplementary material for: Utility of reticulocyte haemoglobin content and percentage hypochromic red cells as markers of iron deficiency anaemia among black CKD patients in South Africa
Source: PLoS One. 2018 Oct 3;13(10):e0204899. doi: 10.1371/journal.pone.0204899 (PMC6169908; doi:10.1371/journal.pone.0204899)
Supplement: S1 Table — (DOC) [file pone.0204899.s002.doc]

Supplementary table 1. Relationship between iron deficiency anaemia and a combination of reticulocyte haemoglobin content and percentage hypochromic red cells among chronic kidney disease participants

| **Factors** | **OR** | **95% CI** | **P-value** |
| --- | --- | --- | --- |
| **CHr** |  |  |  |
| ≤ 28 (ID) | 1.00 | Ref | Ref |
| >28 (NID) | 0.20 | 0.10 - 0.40 | <0.001 |
| **Percentage hypochromic red cells** |  |  |  |
| >5% (ID) | 1.00 | Ref | Ref |
| ≤5% (NID) | 0.72 | 0.38- 1.37 | 0.32 |
| **Gender** |  |  |  |
| Male | 1.00 | Ref | Ref |
| Female | 2.09 | 1.13 - 3.85 | 0.02 |
| **Age group (years)** |  |  |  |
| <25 | 1.00 | Ref | Ref |
| 25 -34 | 0.28 | 0.04 – 1.91 | 0.193 |
| 35 -44 | 0.17 | 0.03 – 0.94 | 0.043 |
| 45 -54 | 0.22 | 0.04 – 1.13 | 0.069 |
| 55 -64 | 0.94 | 0.19 – 4.62 | 0.935 |
| 65 and above | 0.44 | 0.09 – 2.21 | 0.320 |
| **Stage of kidney disease** |  |  |  |
| Early stage | 1.00 | Ref | Ref |
| Late stage | 2.63 | 1.32 -5.22 | 0.006 |
| The model corrected for gender, age, stage of disease. OR: Adjusted odds ratio. Early stage: Stages 1-3; Late stage: Stages 4 and 5.ID: Iron deficiency NID: Non-iron deficiency. AUC of the ROC for model combining ( Chr and %HYPO ) = 0.8123 | | | |
