# Supplementary material for: Utility of reticulocyte haemoglobin content and percentage hypochromic red cells as markers of iron deficiency anaemia among black CKD patients in South Africa
Source: PLoS One. 2018 Oct 3;13(10):e0204899. doi: 10.1371/journal.pone.0204899 (PMC6169908; doi:10.1371/journal.pone.0204899)
Supplement: S2 Table — (DOC) [file pone.0204899.s003.doc]

Supplementary Table 2. Association between Gender and Stage of kidney disease among non-dialysis CKD patients

| **Gender/stage** | **I (n, (%)** | **II (n, (%)** | **III (n, (%)** | **IV n, (%)** | **V (n, (%)** | **Total** | **P-value** |
| --- | --- | --- | --- | --- | --- | --- | --- |
| Male | 6 (26.1) | 20 (58.8) | 48 (57.1) | 30 (50.9) | 30 (51.7) | 134 (51.9) | 0.101 |
| Female | 17(73.9) | 14 (41.2) | 36 (42.9) | 29 (49.2) | 28 (48.3) | 124 (48.1) |
| Total | 23 (100.0) | 34(100.0) | 84(100.0) | 59 (100.0) | 58 (100.0) | 258 (100.0) |
